# Undesirable Levels of Practice Behaviours and Associated Knowledge amongst Community Health Workers in Rural South India Responsible for Type 2 Diabetes Screening and Management

**DOI:** 10.3390/ijerph21050562

**Published:** 2024-04-28

**Authors:** Anirudh G. Gudlavalleti, Giridhara R. Babu, Varun Agiwal, G. V. S. Murthy, Nicolaas C. Schaper, Onno C. P. van Schayck

**Affiliations:** 1 Pragyaan Sustainable Health Outcomes Foundation, World Trade Centre, Nanakramguda, Hyderabad 500032, Telangana, India; president@prasho.org; 2 Department of Family Medicine, Care and Public Health Research Institute (CAPHRI), Maastricht University, P.O. Box 616, 6200 MD Maastricht, The Netherlands; n.schaper@mumc.nl (N.C.S.); onno.vanschayck@maastrichtuniversity.nl (O.C.P.v.S.); 3 Department of Population Medicine, College of Medicine, QU Health, Qatar University, Doha P.O. Box 2713, Qatar; gbabu@qu.edu.qa; 4 Indian Institute of Public Health Hyderabad, Rajendranagar, Hyderabad 500030, Telangana, India; varun.agiwal@iiphh.org

**Keywords:** community health workers, health worker diabetes knowledge, health worker diabetes practice, health worker diabetes behaviour, South-East Asia

## Abstract

Background: Type 2 diabetes (T2DM) poses an enormous global health care challenge, especially among rural communities. Healthcare in these areas can be inadequate and inaccessible due to socio-demographic barriers. To overcome this situation, many low- and middle-income countries are resorting to task shifting, using community health workers (CHWs) for diabetes management. However, its successful implementation depends on the practice behaviours and knowledge of these workers. Objective: This cross-sectional study aimed to evaluate the proficiency of CHWs involved in diabetes screening and management in rural South India by identifying the existing practice behaviours and knowledge gaps. Methods: Employing a customised questionnaire, developed through inputs from experts and government officials, we assessed practice behaviours and the corresponding knowledge base of 275 CHWs. Analytical methodologies consisted of descriptive statistics, logistic regression, and mosaic plots for comprehensive data interpretation. Results: The study showcased significant deficiencies in both practice behaviours (97%) and knowledge (95%) with current mean levels ranging from 48 to 50%, respectively, among the participants. The identified areas of insufficiency were broadly representative of the core competencies required for effective diabetes management, encompassing diabetes diagnosis and referral, HbA1c testing, diabetes diet, diabetes type and self-management, microvascular complications and their screening, peripheral neuropathy management, and diabetes risk assessment. In several areas, correct practice behaviour was reported by a relatively large number of CHWs despite incorrect answers to the related knowledge questions such as referral to the health centres, self-management, and calculation of diabetes risk assessment. Conclusion: This study highlights widespread deficiencies (97% CHWs) in diabetes management practices and knowledge (95% CHWs). To overcome these deficiencies, a thorough needs assessments is vital for effective CHW training. Training of CHWs should not only identify prior knowledge and/or behaviour but also their interrelationship to help create a robust and flexible set of practice behaviours.

## 1. Introduction

Type 2 diabetes mellitus (T2DM) is a pressing global health concern, with an estimated 537 million adults living with the condition worldwide, and 74.2 million cases in India alone [1]. The World Bank data estimates that in 2021, the prevalence of T2DM in Indian adults was 5% [2]. The International Diabetes Federation (IDF) reported that nearly 50% of persons are living with undiagnosed diabetes in India [1]. The report also estimates that this Indian burden is set to grow to 125 million by 2045 significantly affecting a major proportion of its population [1]. Recent trends have indicated the growing prevalence of diabetes amongst lower socioeconomic stratum and rural populations especially in LMICs [3]. With nearly 65% of Indians residing in such areas [4], diabetes poses a huge threat to the population. Prolonged exposure to high blood glucose levels can lead to severe health consequences, particularly micro- and macrovascular complications often leading to morbidity and mortality [1]. An Indian study showed that patients already had some form of diabetic retinopathy at the time of their eye clinic visits, with a potential for blindness if left untreated [5]. Thus, detection and management of T2DM in these areas is extremely critical but is a challenge due to factors like limited access to healthcare services especially in rural areas, lack of trained personnel, unsatisfactory health-seeking behaviour, and long travel distances [3,6,7,8]. Therefore, healthcare systems must develop innovative service models to overcome these barriers.

Task shifting of certain duties of medically trained healthcare workers in rural areas to frontline community health workers (CHWs) is one of the methods used to improve the accessibility of care and reduce the burden on the more qualified health workforce [9]. This approach is being carried out by several developing countries [10,11,12,13,14,15]. In India, multipurpose health workers/assistants and accredited social health activists, typically members of the local communities, are nowadays involved in task shifting in various national health programs for the prevention and management of diabetes, cardiovascular disease, cancer, and stroke [10,16,17,18,19,20]. However, before entrusting the CHWs with the management of the entire spectrum of diabetes screening and management, along with the medical officers, we need to ascertain their capabilities in carrying out the prescribed duties. Evidence reveals a lack of knowledge among these health workers [21,22,23] and we need to better understand what these gaps in knowledge and associated practice behaviours are.

The current study was undertaken to define the current practice behaviours of CHWs responsible for screening and managing diabetes in rural South India and to determine if these behaviours were associated with specific gaps in knowledge. These data would be used as the starting point for the development of a specific training program tailored to the needs of these healthcare workers.

Hence, the research question of this study is as follows: what are the existing gaps in the practice behaviours and knowledge of the CHWs involved in the screening and management of diabetes in rural South India?

## 2. Methodology

This study had a cross-sectional design and included the CHWs involved in diabetes screening and management at the community level and working in eight healthcare administrative regions of Telangana, South India between August 2021 and March 2022. Telangana has approximately 21,400,000 (21.4 million) rural inhabitants, which is almost 62% of its total population. These workers are primarily tasked with population enumeration, home screening, motivating patients to maintain a healthy lifestyle and attend regular complication screenings, supervising of tasks undertaken by junior cadres, conducting meetings with local essential stakeholders, overseeing the data from their health centre, and sharing the data with the authorities.

We used a questionnaire to assess the practice behaviour of the participants and associated knowledge. To develop this questionnaire, we first interviewed senior government officials and medical officers about the common situations faced by CHWs in the community. In these interviews, the most important practice behaviours of the CHWs were identified, as was the knowledge deemed conditional for these practice behaviours. The questionnaire was developed in both English and Telugu (vernacular language), and consisted of 19 questions. This was then piloted with 10 CHWs not included in the study. Based on the feedback received, it was edited and eventually, the validated questionnaire consisted of 17 questions, 10 on practice behaviours and 7 on associated knowledge, as shown in Appendix A, Table A1 and summarised in Table 1.

We administered the questionnaire to 275 CHWs on paper in a face-to-face manner. Because of the diversity in reading and linguistic skills (due to differences in dialects) the content of the questionnaire was explained by two research assistants during group sessions of approximately 25–30 CHWs per session. After all the queries of the CHWs were answered by the research assistants, participants were asked to fill in the answers individually and arrangements were made to ensure no discussion among the participants during this phase of the meeting was possible. The duration of each session was approximately 3.5–4 h. Each session was presided over by two research associates and eight meetings were held in the region where the CHWs were working. The two research associates were trained beforehand and were provided with a handbook to assist them in their roles.

A digital version of the questionnaire was created for each participant in Microsoft (MS). Access and data were imputed by coding for correct and incorrect answers along with missing values. The imputed data were cross verified by the lead researcher and wherever an error was discovered, those physical forms were accessed again to rectify the error. To ensure the veracity of the data, 10% of participants were contacted telephonically for verification. Once confirmed, the data were saved as an Excel workbook which was exported to STATA version 17 for analysis. For scoring the answers to each question, each correct answer was graded as 1 and a wrong answer, a double answer, or no answer was graded as zero. In the initial phase, we asked the government officials and the doctors involved in the development of the questionnaire for the minimal number of correct answers and they formulated a benchmark of 75% correct answers, which we used as an external benchmark for sufficient practice behaviours and knowledge.

### Statistical Analysis

The data were analysed using mean ± standard deviation, median with interquartile ranges (IQR), and frequencies wherever appropriate. The association between correct practice behaviours and their associated knowledge was explored using logistic regression to better understand the relationship between specific gaps in the practice behaviours and knowledge. Finally, to study the relation of correct practice behaviours with incorrect knowledge we used mosaic plots as graphical representations. These plots are divided into four parts, each part depicts the relationship between participants’ practice behaviours (correct or incorrect) with the participant’s knowledge (correct or incorrect). All the significant results were tabulated and are presented in the results section. More details on the plots and the relationships can be found in b.

## 3. Results

All participants (n = 275) filled in the questionnaire; all were females with a mean age of 38.0 + 7.1 years. A majority (approx. 94%) completed high school. The median (IQR) work experience was 10 years (6–15 years). The median overall number of correct answers was 8 (7–10) out of 17 questions. Only 11 participants (4%) crossed the 75% external benchmark of correct answers (2 participants had 14 and 9 participants had 13 correct answers).

In the practice behaviour questionnaire, the median overall number of correct answers was 5 (IQR 4–6) out of 10; only 9 participants (3.3%) crossed the 75% benchmark. The correctly answered percentages for each of the questions are depicted in Table A2 of the electronic supplement. The participants reported sufficient practice behaviours for hypoglycemia management (78% had correct answers) and frequency of risk screening (75%) while the score for health centre referral, self-management, and diabetes signs and symptoms were insufficient (9.8% to 51.3%). The detailed question-wise practice behaviour scores for correct answers have been demonstrated in Table A2 of Appendix A.2.

In the knowledge questionnaire, the median overall number of correct answers was 4 (IQR 3–4) out of 7, only 15 participants (5.5%) crossed the 75% benchmark. The correctly answered percentages for each of the questions are depicted in Table A3 in the electronic supplement. The knowledge about diabetes diet (89.5% correct answers) and diabetic retinopathy/hypoglycemia (85.1%) was sufficient. The knowledge about diabetes diagnosis and referral, types and self-management, and microvascular complications and their screening was insufficient, (30.6% to 57.0% correct answers). The detailed question-wise knowledge scores for correct answers have been demonstrated in Table A3 of Appendix A.3.

In order to study the association between practice behaviour with knowledge, their overall mean scores were standardised to a scale of 10 with overall mean scores of 5.5 and 4.8, respectively. Practice behaviour was associated with knowledge with an odds ratio of 1.64 and a standardised regression coefficient (beta) of 0.50 with a *p*-value < 0.01. Subsequently, for each pair of questions, we examined the association between practice behaviour and knowledge to determine to which extent specific practice behaviour was associated with knowledge on this topic. In Table 2, the associations with a *p* value below 0.2 are shown. The highest odds ratio (1.99) was observed for the association of microvascular complication screening with the knowledge question on this topic (beta-coefficient of 0.68) with a *p*-value of 0.09. This means that having knowledge about the signs and screening of microvascular complications would lead the CHW to practice the correct microvascular screening process 99% more effectively. Similarly, the CHWs were 97% more likely to follow correct practices for peripheral neuropathy management if they had correct knowledge about microvascular complications: signs and screening (odds ratio of 1.97 and beta of 0.68 (*p* = 0.01)). This correct knowledge would also result in the CHWs following the correct practices for identifying the diabetes signs and symptoms in 91% more cases (odds ratio of 1.91 and beta of 0.64 (*p* = 0.01)). The only other pair for which a significant association was observed was for HbA1c testing and diabetes, diagnosis and referral with an odds ratio of only 0.53 and a beta of -0.61 (*p* = 0.04). The detailed correlations between the practice behaviours and the respective knowledge questions are presented in the Table A4, Appendix A.4.

**Figure A1:**
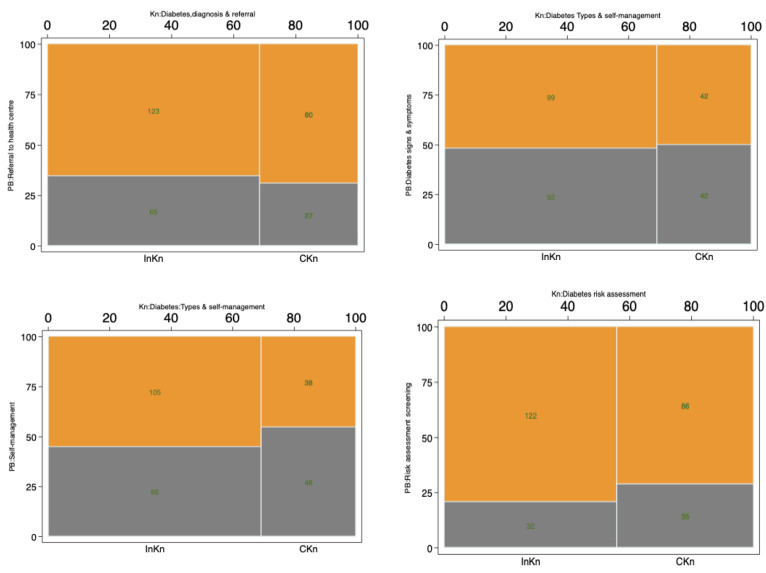
Mosaic Plots Depicting the Strong Relationship between Correct Practice Behaviour and In-correct Knowledge.

We used the mosaic plots to analyse the relationship between correct answers to practice behaviours questions with incorrect knowledge to determine common misconceptions and myths in diabetes care amongst community health workers. There were indeed four such instances where the CHWs correctly answered more than 50% of the practice behaviour questions and incorrectly answered knowledge questions. Table 3 depicts these relationships and their frequencies in detail. The mosaic plots can be found in the Appendix A.5, Figure A1.

## 4. Discussion

The study revealed insufficient practice behaviours and knowledge in ≥95% of CHWs working in rural areas in India and who are responsible for diabetes screening and management. The identified areas of insufficiency were broadly representative of the core competencies required for effective diabetes management, encompassing diabetes diagnosis and referral, HbA1c testing, diabetes diet, diabetes type and self-management, microvascular complications and their screening, peripheral neuropathy management, and diabetes risk assessment.

Our observations align with the existing literature [24,25,26,27] documenting incorrect practices among CHWs due to limited or insufficient knowledge. Hughes et al. [24] emphasise the lack of knowledge among the healthcare workers as a barrier to utilise them in the screening and management of diabetes. Ferguson and Lemay’s study [25] showcases the necessity of case-based training of health workers and elucidates the wide range of topics which need to be taught. Tripathy et al.’s study [26] elucidates the rise of diabetes in rural parts of the country and thus, the increasing need for health workers in controlling this epidemic. Rendrayani et al.’ systematic review [27] reveals the small percentage of studies (less than 30%) showcasing considerable knowledge and positive attitudes towards diabetes management. This underscores the need for thorough needs assessments to identify existing knowledge gaps and incorrect practices among CHWs. By addressing these gaps, we can enhance the effectiveness of CHW training programs and ultimately improve diabetes management outcomes.

Our results showed that the practice behaviours for hypoglycemia management and frequency of risk screening were above desired levels (>75%). The CHWs are often managed with hypoglycemic patients in their course of work within their communities and thus are aware of its management. Similarly, the CHWs receive periodic intimation about screening at-risk individuals from the local governments and thus we see these practices as satisfactory. On the other hand, they have limited training or intimation about referring individuals to health centres, how to promote self-management in patients with diabetes and identifying patients presenting with symptoms of diabetes as this happens most commonly at the health centres and with a doctor. We also find similar results in the knowledge domain, where CHWs are well versed in diet and hypoglycemia/diabetic retinopathy management. This is due to them being trained in these topics, due to various government initiatives. But their deficient knowledge about diagnosis referral, types of diabetes and microvascular complications underscores that their training does not emphasise on these topics and needs to be revised to include these.

The summary score of knowledge revealed a stronger association (OR 9.03, *p* = 0.09) with the summary score of practice behaviours compared to associations of knowledge with practice behaviour when each pair of questions was analysed separately (highest OR 1.99, *p* = 0.09). This observation suggests that while overall knowledge levels tended to positively influence practice behaviours, the relationship was not as evident at the level of specific questions. To further investigate this discrepancy, we employed mosaic plots, which provided insights into potential associations between correct practice behaviours as reported by the respondents and incorrect knowledge. Interestingly, we identified several instances where CHWs reported correct practice behaviours despite possessing incorrect knowledge. This could be attributed to several factors, including prior training, educational differences, or the non-essential nature of specific knowledge for certain practices after adequate training. However, it is crucial to note that even in these cases, incorrect knowledge may hinder the long-term sustainability of the observed correct practice behaviours as it is more likely to push forth incorrect or out-of-date practice behaviours. Thus, all future educational material developed for the health workers related to diabetes screening and management should refrain from focusing on only knowledge or practice. Both of them should be presented as case-based scenarios while integrating the findings of our study. Focusing on only one will yield in incomplete understanding by the CHWs, often leading to either incorrect practices in the field or dissemination of incorrect knowledge amongst the community, causing more harm than benefit. For example, Hughes et al. [24] reported that CHWs in South Africa believed that the main cause for diabetes was higher sugar consumption, due to either poor knowledge or poor knowledge dissemination. In our case, we see that the scores for practices in identifying signs and symptoms for diabetes are low, this would mean that the CHWs might know what these are but may miss out on identifying them when presented by the patient. It is also possible that knowledge regarding diet alone might cause problems, with the CHWs advocating the removal of a macronutrient from the patient’s diet, like carbohydrates, despite this not being a plausible solution in practice. We also see that the CHWs know how to proceed with screening for diabetic retinopathy, but due to their deficient knowledge about peripheral neuropathy, they will not be able to direct any patient to the doctor for any foot-related issue which might result in a gangrene for the patient. Overall, focusing on knowledge or practice in a case-based manner will help the training achieve the end objective of an CHW workforce effective in task shifting for diabetes screening and management.

The major strengths of this study were that the questionnaire was developed in close cooperation with the medical staff ultimately responsible for diabetes care, as well as using input and feedback from the target group studied. We were therefore able to develop the questionnaire into an accessible tool that could be readable and understandable for all CHWs. In addition, we recruited a relatively large number of participants from multiple rural areas, increasing the external validity of our observations. The study also has high generalisability in similar geographies of rural Southeast Asia which bear similarities to our study site like similar diversities of linguistics, education, cultures, practices, and socio-demographic factors. Given the time necessary to explain and subsequently fill in the questionnaire, we had to restrict the number of questions for each domain. Moreover, in a few cases, we had to combine two questions into one question, which might have caused difficulty in comprehension for the CHWs and which may have weakened the associations between knowledge and practice behaviour for a specific topic.

The limitations of the study mainly include the data collection tool’s complexity, wherein the questions were composite in nature and the answers were unable to correctly point out the correlation between the practice behaviour and the knowledge. For further studies, the tool should be further simplified in order to interpret the correlations better.

Finally, some areas require further exploration, like the correct practice behaviours linked with the incorrect practice behaviours, and vice versa; the barriers and enablers for such relationships also need exploration. The analysis surprisingly discovered that the use of HbA1c testing in daily practice was negatively associated with knowledge of diabetes, diagnosis, and referral, with an odds ratio of 0.53, beta-coefficient of 0.61, and *p*-value 0.04. This finding, which is currently difficult to explain, needs further exploration in order to improve future training.

## 5. Conclusions

To conclude, the vast majority of the CHWs involved in diabetes screening and management in rural areas reported inadequate practice behaviour and had insufficient knowledge underlying these behaviours. Defining these gaps in knowledge and practice behaviour is very informative in setting up tailored education programs. However, in several areas, correct practice behaviour was reported by a relatively large number of CHWs despite incorrect answers to the related knowledge questions. Hence, training of CHWs, should not only identify prior knowledge and/or behaviour, but also their interrelationship, to help create a robust and flexible set of practice behaviours that are in line with the guidelines on diabetes care, which are continuously changing over time.

## Figures and Tables

**Table 1:** The associations of practice behaviour with knowledge in the questionnaire (complete questionnaire in Appendix A.1). Bold text indicates a correct answer.

Practice Behaviour & Related Knowledge Questions with Correct Answers in Bold	
Practice Behaviour Questions	Knowledge Questions
PbQ1: You encounter a 40-year-old individual who is 5 feet 4 inches tall and weighs 87 kg. This person reports the following concerns: 1. Frequent urination 2. Excessive thirst 3. Increased appetite 4. Unexplained rapid weight loss What guidance or recommendations would you offer in response to these symptoms?: Answer options: **(1). Refer to the nearest health centre for random blood glucose/HbA1c testing** (2). Refer to the nearest health centre for blood pressure testing (3). Refer to the nearest health centre for thyroid hormone tests (4). None of the above	KnQ1: A 40-year-old woman has a condition where her body doesn’t make enough insulin. She experiences more urination at night, and increased hunger, and thirst. What’s this condition called, and what can she do to feel better? Answer Options (1). Type 1 Diabetes; only exercise **(2). Type 2 Diabetes; exercise, medication & diet control with medicines** (3). Gestational Diabetes; do nothing (4). Monogenic Diabetes; only medication This knowledge question, KnQ1 is associated with practice behaviour questions: PbQ1 and PbQ3
PbQ2: How would you approach the assessment of blood glucose control for a 38-year-old patient with diabetes who refrains from regular blood glucose checks due to a fear of multiple pricks? Answer Options: (1). Advise Fasting blood glucose test at the nearest facility **(2). Advise Glycosylated Hemoglobin- HbA1c test at the nearest facility** (3). Advise Random blood glucose test at the nearest facility (4). Both 1. and 2	KnQ2: Which of these signs indicate diabetes, how is it diagnosed, and where should someone go for treatment? Answer Options (1). Increased Blood Glucose levels, diagnosed using HbA1c and managed at Primary Health Centre (PHC) (2). Increased Blood pressure, diagnosed using HbA1c and managed at PHC (3). Random blood glucose values above or equal to 200mg/dl, diagnosed using Random or fasting glucose and managed at PHC **(4). Both 1. and 3** This knowledge question, KnQ2 is associated with practice behaviour questions: PbQ2 and PbQ7
PbQ3: You have a 48-year-old patient who has had diabetes for three years. They have given you their blood sugar readings from the past two months, and most of them are consistently over 200 mg/dL. What would you suggest to help this patient improve their blood glucose control? Answer Options (1). Regular exercise for at least 30 min for five days a week (2). Consuming a diet rich in proteins (3). Reducing or eliminating habits like smoking, alcohol, tobacco chewing, etc **(4). All of the above**	KnQ3: Which of the following organs or body parts is affected by uncontrolled Diabetes and what is an unexpected fall in blood glucose called Answer Options (1). Stomach; Hyperglycemia **(2). Retina; Hypoglycemia** (3). Both the above are correct (4). None of the above are correct This knowledge question, KnQ3 is associated with practice behaviour questions: PbQ4
PbQ4: A 57-year-old diabetic patient, on insulin for 5 years, experiences symptoms like sweating, tremors, hunger, a fast heartbeat, dizziness, and confusion at night. If his wife calls you for help, what would you advise her to do immediately to improve his condition? Answer Options: (1). Advise the patient to take more Insulin **(2). Advise the patient to take glucose or sugar dissolved in water immediately** (3). Advise the patient to sleep and it will be fine in the morning (4). Will not give any advice to the patient	KnQ4: A 47-year-old man, diabetic for 15 years, reports tingling and burning sensations in his feet. What could this be, and how often should he get checked for it? Answer Options (1). Hypoglycemia; Checked once every year **(2). Peripheral Neuropathy; Checked once every** year (3). Diabetic Ketoacidosis; Checked twice every year (4). Diabetic Retinopathy; Checked twice every year This knowledge question, KnQ4 is associated with practice behaviour questions: PbQ5 and PbQ6
PbQ5: You’re overseeing the care of a 65-year-old diabetic patient. How frequently should you remind her to undergo screening for diabetes-related chronic complications of the eye & kidneys? What should you advise to prevent/manage COVID-19? Answer Options (1). Once every 2 years/Exercise (2). Once every 3 months/COVID Medicine **(3). Once every year (Annually)/COVID-vaccination** (4). Once every 3 years/Nothing can be done	KnQ5: What will you advise for a Diabetic patient during the COVID-19 Pandemic? Answer Options (1). To maintain blood glucose levels within the prescribed range (< 140 mg/dl) (2). To keep a stock of all important medicines (3). To consult a doctor if they have any flu-like symptoms **(4). All of the above** This knowledge question, KnQ5 is associated with practice behaviour questions: PbQ5
PbQ6: A 69-year-old farmer, who has had diabetes for 10 years, had a burning sensation in his feet for a few weeks. He received treatment at a nearby clinic. What advice would you provide to ensure this problem doesn’t come back? Answer Options (1). To be barefoot all the time **(2). To wash and inspect feet daily** (3). To use boiling water to clean feet (4). To leave the feet dry	KnQ6: What foods would you recommend for individuals with diabetes or those at risk of developing diabetes? Answer Options (1). Samosas (Indian fried snack) (2). Bajji/Pakodas (Indian Fritters) **(3). Black Chana (Black Grams)** (4). Laddoos (Indian Dessert) This knowledge question, KnQ6 is associated with practice behaviour questions: PbQ8
PbQ7: Your friend is upset and tells you that her 71-year-old diabetic mother, who has been on medication for 12 years, is experiencing dizziness, burning sensations in her feet, overall tiredness, and easy fatigue. She thinks the medicines aren’t working and asks for your help. What can you do to assist her mother? Answer Options (1). Change her medicine based on her blood glucose values (2). Send her to the ANM to get her medicines changed/modified **(3). Refer her to the Medical Officer at the nearest health centre to change her medicines if needed** (4). Ask her to change her medicines as per the pharmacist’s advise	KnQ7: What does a score below four in the Community Based Assessment Checklist (CBAC)* Assessment mean, and what should be done if the score is less than four? Answer Options (1). The person is free from Non-Communicable (Diseases (NCDs) (2). The person is at higher risk of getting NCDs **(3). The person still needs to be screened for NCDs annually** (4). The person may not attend the weekly NCD Meeting This knowledge question, KnQ7 is associated with practice behaviour questions: PbQ9 & PbQ10
PbQ8: Your patient, aged 46 with diabetes, indulges in sweets and sugarcane juice. Which food can you recommend for satisfying their sweet cravings naturally without impacting their diabetes? Answer Options (1). Mangoes (2). Chikus/Sapotas (3). Grapes **(4). Apples**	
PbQ9: 53-year-old man, who does not move around much, has a family history of diabetes, a waist size over 100 cm, and smokes while drinking alcohol daily. When using the Community Based Assessment Checklist (CBAC), what will be his total score? Answer Options **(1). Seven** (2). Fifteen (3). Four (4). Ten	
PbQ10: As a Community Health Worker (CHW) doing health checks in your village. A 41-year-old woman comes up to you, saying she does not have a history of diabetes or high blood pressure, and she doesn’t seem to have any symptoms of these conditions. What should you do in this situation? Answer Options **(1). Screen the person every year (annually)** (2). No need to screen the person again (3). Screen the person after 2 years (4). Screen the person every 5 years	

**Table 2:** Logistic regression between practice behaviour and knowledge questions.

Practice Behaviour Question	Knowledge Question	Odds Ratio	Coefficient	*p*-Value
HbA1c Testing	Diabetes, Diagnosis and referral	0.53	−0.61	0.04
Self-Management	Diabetes: Types and Self-Management	0.67	−0.39	0.13
Microvascular Complication Screening	Microvascular Complications: Signs and Screening	1.99	0.68	0.09
Peripheral Neuropathy Management	Microvascular Complications: Signs and Screening	1.97	0.68	0.01
Risk Assessment Screening	Diabetes Risk Assessment	0.64	−0.43	0.12
Identifying Diabetes Signs and Symptoms	Microvascular Complications: Signs and Screening	1.91	0.64	0.01

**Table 3:** Relationship of incorrect knowledge and correct practice behaviours among CHWs.

Knowledge	Practice Behaviour	Correct Practice Behaviours Despite Incomplete/Incorrect Knowledge Responses
Knowledge about referral criterion and pathway	Practice Behaviour about Referral to the Health Centre	123 out of 183 participants (67.2%) displayed correct practice behaviour despite incomplete knowledge responses
Knowledge about types of Diabetes	Practice Behaviour about Self-Management	105 out of 143 participants (73.4%) displayed correct practice behaviour despite incomplete knowledge responses
Knowledge about types of diabetes	Practice Behaviour about identifying type 2 diabetes signs and symptoms	99 out of 141 participants (70.2%) displayed correct practice behaviour despite incomplete knowledge responses
Knowledge about diabetes risk assessment	Risk Assessment Screening	122 out of 208 participants (58.7%) displayed correct practice behaviour despite incomplete knowledge responses

## Data Availability

The anonymised data will be available on request for those interested due to ethical considerations.

## Appendix A

### Appendix A.1. Questionnaire Used for Data Collection

**Table A1:** Questionnaire Used for Data Collection.

General Information of CHWs		
Name of CHW		Name of PHC
Age of CHW		Designation of CHW
Gender of CHW		Educational Qualification of CHW
Marital Status of CHW		Work Experience of CHW (in Yrs)
Address of VHW (village Name)		Diatance of residence from PHC
Practice Behaviour & Knowledge Assessment		
Question	Answer Options	
Q1. Which of these signs indicate diabetes, how is it diagnosed, and where should someone go for treatment?	1. Increased Blood Glucose levels, diagnosed using HbA1c and managed at Primary Health Centre (PHC) 2. Increased Blood pressure, diagnosed using HbA1c and managed at PHC 3. Random blood glucose values above or equal to 200 mg/dL, diagnosed using Random or fasting glucose and managed at PHC 4. Both 1. and 3.	
Q2. A 40-year-old woman has a condition where her body doesn’t make enough insulin. She experiences more urination at night, and increased hunger, and thirst. What’s this condition called, and what can she do to feel better?	(1). Type 1 Diabetes; only exercise (2). Type 2 Diabetes; exercise, medication & diet control with medicines (3). Gestational Diabetes; do nothing (4). Monogenic Diabetes; only medication	
Q3-A 38-year-old patient with diabetes is under your care. However, he does not get his blood glucose levels checked regularly due to a fear of multiple pricks. What will you do in this case to get to know his blood glucose control?	1. Advise Fasting blood glucose test at the nearest facility 2. Advise Glycosylated Hemoglobin-HbA1c test at the nearest facility 3. Advise Random blood glucose test at the nearest facility 4. Both 1. and 3.	
Q4-A 65-year-old known diabetic is under your care. How often will you recommend herself be screened for diabetes-associated chronic complications? What should you advise to prevent/manage COVID-19?	(1). Once every 2 years/Exercise (2). Once every 3 months/COVID Medicine (3). Once every year (Annually)/COVID-vaccination (4). Once every 3 years/Nothing can be done	
Q5-A 57-year-old diabetic patient under your care has been on insulin for 5 years. Suddenly at night, his wife calls to tell you that he is suffering from Sweating, Tremors, Hunger, Fast heartbeat, Dizziness, Confusion What will you advise the wife to improve the patient’s condition immediately	1. Advise the patient to take more Insulin 2. Advise the patient to take glucose or sugar dissolved in water immediately 3. Advise the patient to sleep and it will be fine in the morning 4. Will not give any advice to the patient	
Q6-A 47-year-old male with diabetes for 15 years comes to you with a complaint of tingling sensations in the feet and sometimes a burning sensation in the feet. What could this be, and how often should he get checked for it?	(1). Hypoglycemia; Checked once every year (2). Peripheral Neuropathy; Checked once every year (3). Diabetic Ketoacidosis; Checked twice every year (4). Diabetic Retinopathy; Checked twice every year	
Q7-Your friend has been troubled over the past few days and approaches you in tears. She tells you that her 71-year-old mother who has been a diabetic for 12 years and is on medicines has been experiencing dizziness, burning sensation in her feet, overall lethargy and getting tired easily. She thinks her mother’s medicines are not having any effect and wants your help. What can you do to help her mother?	1. Change/Modify her medicine based on her blood glucose values 2. Send her to the ANM to get her medicines changed/modified 3. Refer her to the Medical Officer at the nearest health centre 4. Ask her to change/modify her medicines as per the pharmacist’s advise	
Q8-Which of the following foods will you advise for any person with diabetes or at risk of developing diabetes?	1. Samosas 2. Bajjis/Pakodas 3. Black Chana 4. Laddoos	
Q9-A 46-year-old patient with diabetes who is under your care is constantly taking sweets and sugarcane juice. What can you suggest as a natural alternative to satisfy their sweet tooth?	1. Mangoes 2. Chikus/Sapotas 3. Grapes 4. Apples	
Q10-A 48-year-old patient with diabetes for the last 3 years has come to you with his blood glucose readings for the past two months. You observe that most of his readings are over 200 mg/dl. In such case what can you advise the patient to help them improve their glucose control?	1. Regular exercise for at least 30 min for five days a week 2. Consuming a diet rich in proteins 3. Reducing or eliminating habits like smoking, alcohol, tobacco chewing, etc 4. All of the above	
Q11-What will you advise for a Diabetic patient during the COVID-19 Pandemic?	1. To maintain blood glucose levels within the prescribed range (<140 mg/dL) 2. To keep a stock of all important medicines 3. To consult a doctor if they have any flu-like symptoms 4. All of the above	
Q12-Which of the following organs or body parts is affected by uncontrolled Diabetes and what is an unexpected fall in blood glucose called	(1). Stomach; Hyperglycemia (2). Retina; Hypoglycemia (3). Both the above are correct (4). None of the above are correct	
Q13-A 69-year-old farmer who has been diabetic for the last 10 years has been complaining to you about a burning sensation in his feet for the last couple of weeks. You sent him to the nearest health facility where the doctor treated it. Now what should be your advice to ensure that this condition does not arise for the patient again?	1. To be barefoot all the time 2. To wash and inspect feet daily 3. To use boiling water to clean feet 4. To leave the feet dry	
Q14-A 40-year-old person, with a height of 5‘4 and weight of 87 kgs presents to you with the following problems: Frequent urination, Excessive thirst Increased hunger & Sudden weight loss. What would you advise?	1. Refer to the nearest health centre for random blood glucose testing/HbA1C testing 2. Refer to the nearest health centre for blood pressure testing/ 3. Refer to the nearest health centre for thyroid hormone tests 4. None of the above	
Q15-A 53-year-old male who leads a sedentary lifestyle with a family history of Diabetes, a waist circumference of more than 100 cm and habits of smoking, and consuming alcohol daily has undergone CBAC Assessment. What will be the total score?	1. Seven 2. Fifteen 3. Four 4. Ten	
Q16-What does a score less than four in the CBAC Assessment imply?	1. The person is free from NCDs 2. The person is at higher risk of getting NCDs 3. The person still needs to be screened for NCDs annually 4. The person may not attend the weekly NCD Meeting	
Q17-You are a part of the NCD screening in your village this year. A 41-year-old female comes to enquire about what is happening. On further interaction, you learn that she has no history of diabetes or hypertension and also does not share any signs/symptoms of either. In such a case what will be your plan of action?	1. Screen the person every year (annually) 2. No need to screen the person again 3. Screen the person after 2 years 4. Screen the person every 5 years	

### Appendix A.2. Question-Wise Practice Behaviour Scores of the Community Health Workers

**Table A2:** Question-Wise Practice Behaviour Scores of the Community Health Workers.

Practice Behaviour Scores			
Themes	Questions	Percentage of Participants with Correct Answers % (n)	Confidence Intervals
Practice behaviour	Diabetes signs and symptoms	51.3% (141)	45–57%
	Referral to health centre	66.5% (183)	60–72%
	HbA1c testing	31.6% (87)	26–37%
	Diabetes diet	41.8% (115)	35–47%
	Self-management	52% (143)	45–58%
	Hypoglycemia management	78% (215)	72–82%
	Microvascular complication screening	9.8% (27)	6–13%
	Peripheral neuropathy management	29.5% (81)	24–35%
	Calculation of diabetes risk score	26.6% (73)	21–32%
	Frequency risk screening	75.6% (208)	70–80%

### Appendix A.3. Question-Wise Knowledge Scores of the Community Health Workers

**Table A3:** Question-Wise Knowledge Scores of the Community Health Workers.

Knowledge Score			
Themes	Questions	Percentage of Participants with Correct Answers % (n)	Confidence Intervals
Knowledge questions	Diabetes, diagnosis and referral	31.6% (87)	26–37%
	Diabetes: types and self-management	30.6% (84)	25–36%
	Diabetes diet	89.5% (246)	85–92%
	Diabetic retinopathy and Hypoglycemia (combined question)	85.1% (234)	80–89%
	Microvascular complications: signs and screening	36.7% (101)	31–42%
	Diabetes and COVID-19	57.1% (157)	51–63%
	Diabetes risk assessment	44% (121)	38–50%

### Appendix A.4. Logistic Correlations between Practice behaviours and Knowledge Themes

**Table A4:** Logistic Correlations between Practice behaviours and Knowledge Themes.

Practice Behaviour Theme	Knowledge Theme	Odds Ratio	Coefficient	*p*-Value
HbA1c Testing	Diabetes, Diagnosis and referral	0.53	−0.61	0.04
Diabetes patient referral to health centre	Diabetes, Diagnosis and referral	1.17	0.16	0.56
Self-Management	Diabetes: Types and Self-Management	0.67	−0.39	0.13
Diabetes signs and symptoms	Diabetes: Types and Self-Management	0.92	−0.07	0.77
Identifying signs and symptoms of diabetes	Microvascular Complications: Signs and Screening	1.91	0.64	0.01
Microvascular complications screening	Microvascular Complications: Signs and Screening	1.99	0.68	0.09
Peripheral Neuropathy Management	Microvascular Complications: Signs and Screening	1.97	0.68	0.01
Microvascular complication management: Hypoglycemia	Microvascular complication knowledge: Hypoglycemia & DR	1.6	0.47	0.21
Practice: Diabetes Diet	Diabetes Diet	1.68	0.52	0.21
Risk Assessment Calculation	Diabetes Risk Assessment	0.78	−0.23	0.39
Risk Assessment Screening	Diabetes Risk Assessment	0.64	−0.43	0.12
Overall Practice Behaviour	Overall Knowledge	9.03	0.499	0.09

### Appendix A.5. Mosaic Plots Depicting the Strong Relationship between Correct Practice Behaviour and Incorrect Knowledge

Each mosaic plot consists of four sections. Each plot has the number of participants answering knowledge questions on the *X*-axis and practice behaviour questions on the *Y*-axis. In the plot, there are two columns, each divided into two parts. The left column shows the number of participants who answered knowledge questions incorrectly. The right column shows the same for correctly answered knowledge questions. The upper part of each column shows the number of participants who answered practice behaviour questions correctly and the lower part who answered incorrectly. Further, the upper right section shows the number of participants who answered both knowledge and practice behaviour questions correctly, and the upper left section shows the number of participants who answered the practice behaviour questions correctly but the knowledge questions incorrectly. The lower left section shows the number of participants who answered both the knowledge and practice behaviour questions incorrectly. The lower right section shows the number of participants who answered knowledge questions correctly but practice behaviour questions incorrectly.
